# Phase 3, randomized, open-label study of pembrolizumab plus lenvatinib versus chemotherapy for first-line treatment of advanced or recurrent endometrial cancer: ENGOT-en9/LEAP-001

**DOI:** 10.1136/ijgc-2021-003017

**Published:** 2021-11-19

**Authors:** Christian Marth, Rafal Tarnawski, Alexandra Tyulyandina, Sandro Pignata, Lucy Gilbert, Diego Kaen, M Jesús Rubio, Sophia Frentzas, Mario Beiner, Manuel Magallanes-Maciel, Laura Farrelly, Chel Hun Choi, Regina Berger, Christine Lee, Christof Vulsteke, Kosei Hasegawa, Elena Ioanna Braicu, Xiaohua Wu, Jodi McKenzie, John J Lee, Vicky Makker

**Affiliations:** 1 AGO-Austria and Department of Obstetrics and Gynaecology, Medical University of Innsbruck, Innsbruck, Austria; 2 Maria Sklodowska-Curie National Research Institute of Oncology Gliwice Branch abd PGOG, Gliwice, Poland; 3 CHRU Brest, Brest, France; 4 NN Blokhin Russian Cancer Research Centre and IM Sechenov First Moscow State Medical University (Sechenov University), Moscow, Russian Federation; 5 Department of Urology and Gynecology, Istituto Nazionale Tumori IRCCS Fondazione G Pascale and MITO, Napoli, Italy; 6 McGill University Health Centre, Montreal, Quebec, Canada; 7 Centro Oncologico Riojano Integral and National University of La Rioja, La Rioja, Argentina; 8 H Reina Sofía de Córdoba and GEICO Group, Córdoba, Spain; 9 Monash Health and Monash University, Melbourne, Victoria, Australia; 10 Meir Medical Center and ISGO, Kfar Saba, Israel; 11 Centro Oncológico Internacional, Mexico City, Mexico; 12 Cancer Research UK and University College London Cancer Trials Centre, Cancer Institute, University College London and NCRI, London, UK; 13 Samsung Medical Center, Seoul, The Republic of Korea; 14 AGO-Austria and University Hospital for Gynaecology and Obstetrics, Medical University of Innsbruck, Innsbruck, Austria; 15 Texas Oncology - Woodlands, The Woodlands, Texas, USA; 16 Department of Medical Oncology, Integrated Cancer Center Ghent, AZ Maria Middelares Ghent and Center of Oncological Research, Integrated Personalized and Precision Oncology Network, University of Antwerp and BGOG, Wilrijk, Belgium; 17 Saitama Medical University International Medical Center, Hidaka, Saitama, Japan; 18 Charité Universitätsmedizin Berlin and North Eastern German Society for Gynecologic Oncology (NOGGO), Berlin, Germany; 19 Fudan University Shanghai Cancer Center, Shanghai, China; 20 Eisai Inc, Woodcliff Lake, New Jersey, USA; 21 Merck & Co Inc, Kenilworth, New Jersey, USA; 22 Memorial Sloan-Kettering Cancer Center and Weill Cornell Medical Center, New York, New York, USA

**Keywords:** uterine cancer, endometrial neoplasms

## Abstract

**Background:**

Pembrolizumab plus lenvatinib is a novel combination with promising efficacy in patients with advanced and recurrent endometrial cancer. This combination demonstrated high objective response rates in a single-arm phase 1b/2 trial of lenvatinib plus pembrolizumab in patients with advanced endometrial cancer (KEYNOTE-146/Study 111) after ≤2 previous lines of therapy. In a randomized phase 3 trial of lenvatinib in combination with pembrolizumab versus treatment of physician's choice in patients with advanced endometrial cancer (KEYNOTE-775/Study 309), after 1‒2 previous lines of therapy (including neoadjuvant/adjuvant), this combination improved objective response rates, progression-free survival, and overall survival compared with chemotherapy.

**Primary Objective:**

To compare the efficacy and safety of first-line pembrolizumab plus lenvatinib versus paclitaxel plus carboplatin in patients with newly diagnosed stage III/IV or recurrent endometrial cancer, with measurable or radiographically apparent disease.

**Study Hypothesis:**

Pembrolizumab plus lenvatinib is superior to chemotherapy with respect to progression-free survival and overall survival in patients with mismatch repair-proficient tumors and all patients (all-comers).

**Trial Design:**

Phase 3, randomized (1:1), open-label, active-controlled trial. Patients will receive pembrolizumab intravenously every 3 weeks plus lenvatinib orally daily or paclitaxel plus carboplatin intravenously every 3 weeks, stratified by mismatch repair status (proficient vs deficient). Patients with mismatch repair-proficient tumors will be further stratified by Eastern Cooperative Oncology Group performance status (0/1), measurable disease (yes/no), and prior chemotherapy and/or chemoradiation (yes/no).

**Major Inclusion/Exclusion Criteria:**

Adults with stage III/IV/recurrent histologically confirmed endometrial cancer that is measurable or radiographically apparent per blinded independent central review. Patients may have received previous chemotherapy only as neoadjuvant/adjuvant therapy and/or concurrently with radiation. Patients with carcinosarcoma (malignant mixed Müllerian tumor), endometrial leiomyosarcoma, or other high grade sarcomas, or endometrial stromal sarcomas were excluded.

**Primary Endpoints:**

Progression-free and overall survival (dual primary endpoints).

**Sample Size:**

About 875 patients.

**Estimated Dates for Completing Accrual and Presenting Results:**

Enrollment is expected to take approximately 24 months, with presentation of results in 2022.

**Trial Registration:**

ClinicalTrials.gov, NCT03884101.

## INTRODUCTION

Carcinoma of the uterine corpus, or endometrial cancer, makes up 2% of all new cancer cases,^1^ and for most patients it is typically detected in the early stages when disease is confined to the uterus.^2^ Five-year survival rates are high for patients with early-stage disease, and 5-year survival rates of 76% have been reported for all patients with endometrial cancer in Europe (all disease stages).^3 4^ However, approximately 13% of patients experience recurrent disease.^5^ The standard of care for patients with advanced or recurrent disease is multiagent systemic chemotherapy, including paclitaxel plus carboplatin in the first-line setting.^6^ There is an urgent need to provide treatment options that yield better outcomes because the prognosis for these patients remains poor, with a 5-year survival rate of 17% in the recurrent metastatic setting.^2^

The combination of pembrolizumab and lenvatinib has emerged as an effective treatment for advanced, previously treated endometrial cancer. Pembrolizumab is an anti-programmed death 1 monoclonal antibody that blocks the interaction between programmed death 1 and programmed death ligands 1 and 2, and has been shown to be effective in the treatment of a variety of solid tumor types, including mismatch repair-deficient endometrial cancer.^7 8^ Lenvatinib is a selective inhibitor of vascular endothelial growth factor receptors 1–3 and other receptor tyrosine kinases, such as fibroblast growth factor receptors 1–4, platelet-derived growth factor receptor α, KIT, and RET, and is a potent angiogenesis inhibitor.^9^ It has also been shown to be an effective immunomodulator. Preclinical models have shown that lenvatinib decreases tumor-associated macrophages, increases T-cell populations, upregulates the type I interferon signaling pathway, and leads to activation of CD8-positive T cells. In preclinical models, lenvatinib in combination with anti-programmed death 1 therapy significantly suppressed and delayed tumor growth compared with either treatment alone.^9^

In a single-arm, phase 1b/2 trial of lenvatinib plus pembrolizumab, KEYNOTE-146/Study 111,^10^ the combination of pembrolizumab and lenvatinib was associated with an objective response rate of 39.5% in patients with previously treated endometrial cancer (irrespective of mismatch repair status).^10^ In a randomized phase 3 trial of lenvatinib in combination with pembrolizumab versus treatment of physician's choice in patients with advanced endometrial cancer (KEYNOTE-775/Study 309),^11^ patients who had received one or two previous platinum based chemotherapy regimens (including in the neoadjuvant or adjuvant setting) had significantly prolonged progression-free and overall survival with pembrolizumab plus lenvatinib compared with physician’s treatment of choice (paclitaxel or doxorubicin) in both patients with mismatch repair-proficient disease (progression-free survival hazard ratio (HR) 0.60; overall survival HR 0.68) and in all enrolled patients (progression-free survival HR 0.56; overall survival HR 0.62).^11^

In the ENGOT-en9/LEAP-001 study (ClinicalTrials.gov NCT03884101; protocol MK-7902-001-05/E7080-G000-313/ENGOT-EN9; dated March 17, 2021), we hypothesize that the combination of pembrolizumab plus lenvatinib is superior to standard of care paclitaxel and carboplatin chemotherapy in the first-line setting with respect to progression-free survival and overall survival in both patients with mismatch repair-proficient endometrial cancer and all-comers (all enrolled patients with mismatch repair-proficient or mismatch repair-deficient tumors). In patients with mismatch repair-proficient endometrial cancer, superiority will be tested after non-inferiority to chemotherapy with respect to overall survival has been evaluated.

## METHODS AND ANALYSIS

### Trial Design

ENGOT-en9/LEAP-001 is a phase 3, randomized, open-label, active-controlled trial of pembrolizumab in combination with lenvatinib compared with platinum doublet chemotherapy (paclitaxel and carboplatin). The first patient was enrolled on April 11, 2019. This study has completed recruitment and was conducted in 190 community clinics and academic hospitals in 22 countries in accordance with Good Clinical Practice guidelines and the Declaration of Helsinki. The study protocol and amendments were approved by institutional review boards or independent ethics committees at each study site. All patients provided written informed consent to the study investigator before undergoing any protocol-specific procedure.

**Figure 1:**
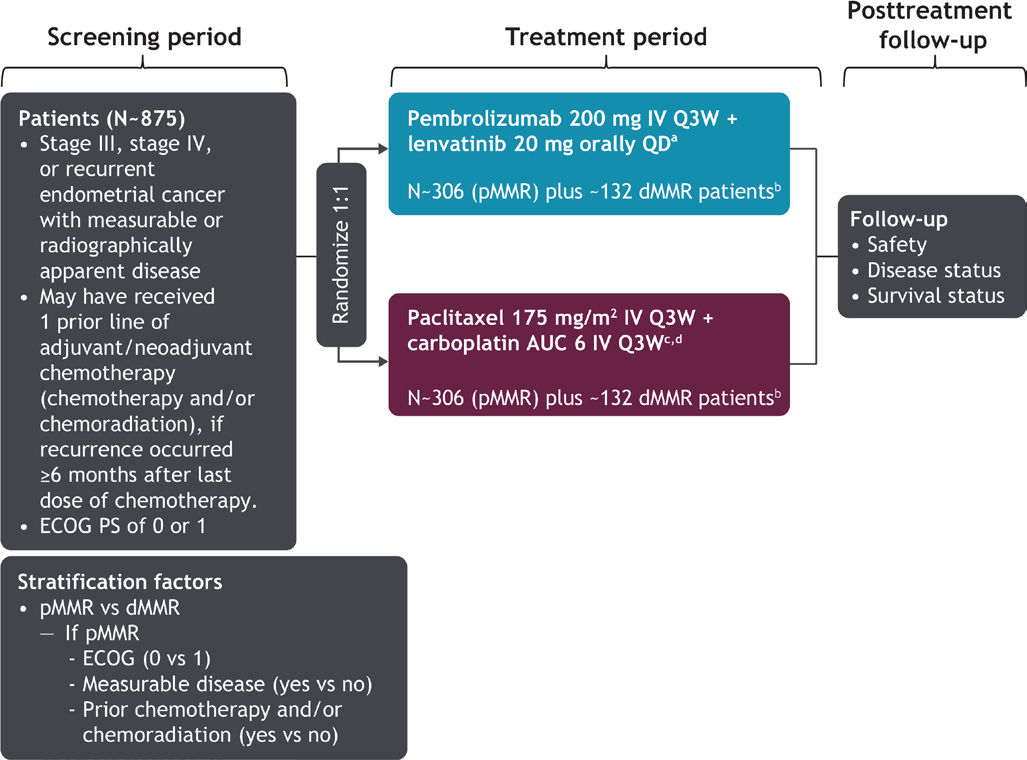
ENGOT-en9/LEAP-001 study design. ^a^Treat until disease progression or unacceptable toxicity. Pembrolizumab must be stopped after 35 cycles, but lenvatinib may continue after stopping pembrolizumab. ^b^Study will be fully enrolled when 612 patients with mismatch repair-proficient (pMMR) tumors and ~263 patients with mismatch repair-deficient (dMMR) tumors are recruited. ^c^A lower starting dose of paclitaxel (135 mg/m^2^) and carboplatin (AUC 5 mg/mL/min) may be administered to patients at risk of developing toxicities due to previous pelvic/spine radiation. An AUC of 5 mg/mL/min dose of carboplatin may be administered in accordance with local practice. ^d^Patients may receive up to seven cycles of paclitaxel/carboplatin; however, chemotherapy treatment beyond seven cycles may be permitted (with the sponsors’ approval) for patients who continue to derive clinical benefit. AUC, area under the curve (unit, mg/mL/min); ECOG, Eastern Cooperative Oncology Group; IV, intravenous; PS, performance status; QD, once daily; Q3W, every 3 weeks.

The study design is shown in Figure 1. Patients were randomly assigned in a 1:1 ratio to receive either pembrolizumab 200 mg intravenously once every 3 weeks in combination with lenvatinib 20 mg orally daily or paclitaxel 175 mg/m^2^ in combination with carboplatin area under the curve 6 mg/mL/min intravenously every 3 weeks. Study treatment was discontinued if patients experienced disease progression or unacceptable toxicity. Pembrolizumab must have been discontinued after 35 cycles, but lenvatinib could have been continued after pembrolizumab discontinuation. Patients could have received up to seven cycles of paclitaxel/carboplatin; however, chemotherapy treatment beyond seven cycles was permitted for patients who continued to derive clinical benefit. Lenvatinib dosing was reduced, interrupted, or discontinued according to protocol-specified guidelines for patients who experienced intolerable grade 2–3 adverse events or any grade 4 adverse events. Lenvatinib compliance was monitored based on the drug accountability documented by the site staff. Treatment for complications or adverse events could have been administered at the investigator’s discretion unless it was expected to interfere with the evaluation of, or interact with, study medication.

Funding and sponsorship for this research was provided by Merck Sharp & Dohme Corp, a subsidiary of Merck & Co Inc (Kenilworth, New Jersy, USA), and Eisai Inc (Woodcliff Lake, New Jersy, USA). The trial is being performed in collaboration with the European Network for Gynaecological Oncological Trial groups (ENGOT), which is a research network of the European Society of Gynaecological Oncology (ESGO).

### Participants

Key patient eligibility criteria are listed in Table 1. Briefly, adults with stage III, IV, or recurrent histologically confirmed endometrial cancer that is measurable or non-measurable per Response Evaluation Criteria in Solid Tumors (RECIST) v1.1, but radiographically apparent as assessed by blinded independent central review, and Eastern Cooperative Oncology Group performance status ≤1 were eligible. Patients may have received prior chemotherapy only as neoadjuvant/adjuvant therapy and/or concurrently with radiation if the recurrence occurred ≥6 months after the last dose of chemotherapy. Patients with carcinosarcoma (malignant mixed Müllerian tumor), endometrial leiomyosarcoma, or other high-grade sarcomas, or endometrial stromal sarcomas, were excluded.

**Table 1:** Key patient eligibility criteria

Key inclusion criteria	Key exclusion criteria
Stage III, IV, or recurrent, histologically confirmed endometrial carcinoma with measurable or radiographically apparent disease* Prior therapies may include chemotherapy (only if administered as neoadjuvant or adjuvant therapy and/or concurrently with radiation), radiation, or hormonal therapy (only if discontinued ≥1 week before randomization) Provided archival tumor tissue or newly obtained biopsy of tumor for determination of mismatch repair status Eastern Cooperative Oncology Group performance status of 0/1	Carcinosarcoma, endometrial leiomyosarcoma, or other high grade sarcomas, or endometrial stromal sarcomas Additional malignancies that have progressed or required active treatment in the last 3 years† Gastrointestinal conditions that might affect absorption of lenvatinib Active infection requiring systemic treatment Previous therapy with any treatment targeting vascular endothelial growth factor-directed angiogenesis; anti-PD-1, anti-PD-L1, or anti-PD-L2 agents; or any agent directed at another stimulatory or co-inhibitory T cell receptor Inadequate organ function

*Disease may be either measurable or non-measurable per Response Evaluation Criteria in Solid Tumors v1.1 but must be radiographically apparent by blinded independent central review.

†Not including basal cell carcinoma of the skin, squamous cell carcinoma of the skin, or carcinoma in situ that has undergone potentially curative therapy.

PD-1, programmed death 1; PD-L1, programmed death ligand 1; PD-L2, programmed death ligand 2.

### Endpoints

The objective of this study was to compare the efficacy and safety of first-line pembrolizumab in combination with lenvatinib versus paclitaxel plus carboplatin in patients with newly diagnosed, stage III, IV, or recurrent endometrial cancer. The dual primary endpoints were progression-free survival per RECIST v1.1 by blinded independent central review (modified to follow a maximum of 10 target lesions and five target lesions per organ) and overall survival in patients with mismatch repair-proficient tumors and in all patients (all-comers). Progression-free survival was defined as the time from randomization to first documented disease progression or death due to any cause, whichever occurred first. Overall survival was defined as the time from randomization to death due to any cause. Patient survival status is assessed routinely, including after disease progression or start of new anticancer therapy. Mismatch repair status was assessed by a central laboratory (Neogenomics, Fort Myers, Florida, USA) by immunohistochemistry (Ventana, Tuscon, Arizona, USA) using archived tumor tissue or a fresh biopsy before randomization.

Secondary endpoints were objective response rate per RECIST v1.1 by blinded independent central review in patients with mismatch repair-proficient tumors and in all-comers, health related quality of life in patients with mismatch repair-proficient tumors and in all-comers, and safety and tolerability in all-comers. Objective response was defined as a confirmed complete or partial response. Health-related quality of life is evaluated using the mean change from baseline in the European Organization for Research and Treatment of Cancer Quality of Life Questionnaire‒Core 30 global health status/quality of life score. Safety and tolerability were assessed by clinical review of adverse events, laboratory tests, vital signs, and ECG measurements throughout the study until 90 days (120 days for serious adverse events) after the last dose of study treatment, or until 30 days after the last dose if new anticancer therapy was initiated.

Key exploratory endpoints included duration of response, disease control rate, and clinical benefit rate per RECIST v1.1 by blinded independent central review in patients with mismatch repair-proficient tumors and in all-comers. Duration of response was defined as the time from first documented response to first documented disease progression or death, whichever occurred first. Disease control was defined as the best overall response of complete or partial response or of stable disease ≥7 weeks after randomization. Clinical benefit was defined as best overall response of complete or partial response or of stable disease with duration ≥23 weeks after randomization.

### Sample Size

Sample size was calculated to allow hypothesis testing of the dual primary endpoints of progression-free survival and overall survival to be well powered. The study is considered to be fully enrolled when 612 mismatch repair-proficient participants have enrolled. At the time of enrollment completion, approximately 875 total participants are expected to be enrolled, with 612 mismatch repair-proficient participants and approximately 263 mismatch repair-deficient participants.

### Randomization and Blinding

Patients were randomized 1:1 to receive pembrolizumab in combination with lenvatinib or to receive paclitaxel in combination with carboplatin. Randomization was conducted using an interactive response technology system. Randomization was first stratified by mismatch repair status (proficient vs deficient) with patients with mismatch repair-proficient tumors further stratified by Eastern Cooperative Oncology Group performance status (0 vs 1), measurable disease (yes vs no), and previous chemotherapy and/or chemoradiation (yes vs no). This was an open-label study and therefore, no blinding was performed.

### Statistical Methods

All randomized patients will be included in the efficacy analysis population and all patients who received ≥1 dose of study treatment will be included in the safety analysis population. The primary endpoints of progression-free and overall survival will be estimated using the non-parametric Kaplan–Meier method. Treatment differences will be assessed using the stratified log rank test, and the magnitude of treatment difference will be estimated using a stratified Cox proportional hazards model with the Efron method of tie handling. The stratification factors used for randomization will be applied to the stratified log-rank test and the stratified Cox model.

## DISCUSSION

Several studies have evaluated the use of anti-programmed death 1 or anti-programmed death ligand 1 monoclonal antibodies as monotherapy in patients with previously treated, advanced, or recurrent endometrial cancer (Table 2), including pembrolizumab,^8 12^ dostarlimab (anti-programmed death 1),^13 14^ durvalumab (anti-programmed death ligand 1),^15^ and avelumab (anti-programmed death ligand 1).^16^ Across these studies, objective response rates ranged between 27% and 57% in patients with mismatch repair-deficient tumors and between 3% and 14% in patients with mismatch repair-proficient tumors.^8 12–16^ Lenvatinib monotherapy was also evaluated in a phase 2 trial of 133 patients with recurrent endometrial cancer with an objective response rate of 14.3% with a manageable safety profile.^17^

**Table 2:** Clinical trials evaluating anti-programmed death 1/programmed death ligand 1 therapies in patients with previously treated endometrial cancer

Study	Phase	Therapy	Disease	Previous therapy	Biomarkers	N	Objective response rate (%)
KEYNOTE-028^12^	1b	Pembrolizumab (anti-PD-1)	Locally advanced or metastatic endometrial cancer	Patients had disease that had progressed after standard therapy, or for which no standard therapy exists, or for which standard therapy is not appropriate	PD-L1-positive	24 (23 in efficacy analysis)	13.0% (RECIST v1.1 by investigator review)
KEYNOTE-158^8^	2	Pembrolizumab (anti-PD-1)	Unresectable and/or metastatic incurable endometrial carcinoma	Patients had disease that had progressed on or was intolerant to previous standard therapy	MSI-H/dMMR	49	57.1% (RECIST v1.1 by independent central radiologic review)
GARNET^14^	1	Dostarlimab (anti-PD-1)	Recurrent or advanced endometrial cancer	Patients had disease that had progressed during or after chemotherapy with ≤2 previous lines of therapy	dMMR	110	45.5% (RECIST v1.1 by blinded independent central review)
					pMMR	144	13.9% (immune-related RECIST by investigator assessment)
PHAEDRA (ANZGOG1601)^15^	2	Durvalumab (anti-PD-L1)	Advanced endometrial cancer	Patients had disease that had progressed after 0–3 previous lines of chemotherapy	dMMR	35	40% (immune-related RECIST)
				Patients had disease that had progressed after 1–3 previous lines of chemotherapy	pMMR	36	3% (immune-related RECIST)
NCT02912572^16^	2	Avelumab (anti-PD-L1)	Recurrent endometrial cancer	Patients had received ≥1 previous lines of chemotherapy	dMMR	15	26.7% (RECIST v1.1)
					pMMR	16	6.25% (RECIST v1.1)
KEYNOTE-146/ Study 111^10^	1b/2	Pembrolizumab + lenvatinib (anti-PD-1 + tyrosine kinase inhibitor)	Advanced endometrial cancer	Patients had received ≤2 previous lines of systemic therapy (≥2 lines allowed at sponsor’s discretion)	dMMR or pMMR	124	39.5% (immune-related RECIST by investigator assessment)
					dMMR	11	63.6% (immune-related RECIST by investigator assessment)
					pMMR	94	36.2% (immune-related RECIST by investigator assessment)
KEYNOTE-775/ Study 309^11^	3	Pembrolizumab + lenvatinib (anti-PD-1 + tyrosine kinase inhibitor) vs doxorubicin or paclitaxel	Advanced endometrial cancer	Patients had received 1‒2 previous platinum-based chemotherapy regimens (including neoadjuvant/adjuvant)	dMMR or pMMR	827	31.9% vs 14.7% (RECIST v1.1 by blinded independent central review)
					pMMR	697	30.3% vs 15.1% (RECIST v1.1 by blinded independent central review)

dMMR, mismatch repair-deficient; MSI-H, microsatellite instability high; PD-1, programmed death 1; PD-L1, programmed death ligand 1; pMMR, mismatch repair-proficient; RECIST, Response Evaluation Criteria in Solid Tumors.

Clinical trials evaluating pembrolizumab plus lenvatinib in patients with endometrial cancer demonstrated objective response rates of 32–40%.^10 11^ Results were similar among patients with mismatch repair-proficient tumors (30‒36%). In a single-arm, phase 1b/2 trial (KEYNOTE-146/Study 111),^10^ this combination was associated with an objective response rate of 39.5% in a cohort of patients with endometrial cancer, most of whom had received previous therapy.^10^ In the KEYNOTE-775/Study 309 trial that evaluated pembrolizumab in combination with lenvatinib compared with physician’s treatment of choice (paclitaxel or doxorubicin chemotherapy), progression-free survival, overall survival, and objective response rate were all significantly improved in the pembrolizumab and lenvatinib arm compared with the physician’s treatment of choice (paclitaxel or doxorubicin chemotherapy) in patients with mismatch repair-proficient tumors (n=697; median progression-free survival 6.6 vs 3.8 months (HR 0.60); median overall survival 17.4 vs 12.0 months (HR 0.68); objective response rate 30.3% vs 15.1% (p<0.0001)) and in all-comers (n=827; median progression-free survival 7.2 vs 3.8 months (HR 0.56); median overall survival 18.3 vs 11.4 months (HR, 0.62); objective response rate 31.9% vs 14.7% (p<0.0001)).^11^ Based on the results of the confirmatory KEYNOTE-775/Study 309 trial, the US Food and Drug Administration accelerated approval for pembrolizumab plus lenvatinib in this setting was converted to a full approval.^7^

Given the positive results from KEYNOTE-146/Study 111 and KEYNOTE-775/Study 309, we are optimistic that the results of the ENGOT-en9/LEAP-001 study will demonstrate a similar clear progression-free survival and overall survival benefit of pembrolizumab in combination with lenvatinib in the first-line treatment of patients with primary advanced or recurrent endometrial cancer. This trial has the potential to define the new standard of first-line treatment in recurrent endometrial cancer. There are also other ongoing trials evaluating programmed death 1/programmed death ligand 1 antibody-based combinations in the first-line setting in patients with primary advanced or recurrent endometrial cancer (Table 3). Data from the ENGOT-en9/LEAP-001 study will be presented at upcoming scientific meetings, submitted to a peer reviewed journal for publication, and posted on trial registries.

**Table 3:** Select ongoing clinical trials evaluating anti-programmed death 1/programmed death ligand 1 therapies in patients with primary advanced or recurrent endometrial cancer

Study	Phase	Therapy	Disease	Previous therapy	Biomarkers	N	Location
ENGOT-en6/NSGO-RUBY^18^ NCT03981796	3	Dostarlimab (anti-PD-1) + carboplatin + paclitaxel followed by dostarlimab with or without niraparib vs placebo + carboplatin + paclitaxel followed by placebo	First recurrent or primary stage III–IV endometrial cancer	Patients had received no previous systemic chemotherapy; previous neoadjuvant/adjuvant chemotherapy permitted if recurrence ≥6 months	MSI-H or MSS	~470	Europe, North America
ENGOT-en7/AtTEnd^19^ NCT03603184	3	Paclitaxel + carboplatin + atezolizumab (anti-PD-L1) followed by atezolizumab vs paclitaxel + carboplatin + placebo followed by placebo	Newly diagnosed, advanced stage III–IV or recurrent endometrial cancer	Patients had received one previous line of chemotherapy if upfront/adjuvant and platinum-free interval ≥6 months	None	~550	Australia, Europe, Japan, New Zealand
ENGOT-en9/LEAP-001 (current study) NCT03884101	3	Pembrolizumab + lenvatinib (anti-PD-1 + tyrosine kinase inhibitor) vs paclitaxel + carboplatin	Stage III, IV, or recurrent endometrial cancer	Patients may have received previous chemotherapy (as neoadjuvant/adjuvant therapy and/or concurrently with radiation) radiation, or hormonal therapy (completed ≥1 week prior)	dMMR or pMMR	~875	Asia, Australia, Europe, North America, South America
NRG-GY018 NCT03914612	3	Pembrolizumab (anti-PD-1) + paclitaxel + carboplatin followed by pembrolizumab vs placebo + paclitaxel + carboplatin followed by placebo	Stage III–IVB or recurrent endometrial cancer	Patients may have received prior adjuvant chemotherapy (completed ≥12 months prior), radiation therapy (completed ≥4 weeks prior), or hormonal therapy (completed ≥3 weeks prior)	PD-L1-positive or negative; dMMR or pMMR	~810	North America

dMMR, mismatch repair-deficient; MSI-H, microsatellite instability high; MSS, microsatellite stable; PD-1, programmed death 1; PD-L1, programmed death ligand 1; pMMR, mismatch repair-proficient.

## Data Availability

Merck Sharp & Dohme Corp., a subsidiary of Merck & Co., Inc., Kenilworth, NJ, USA (MSD) is committed to providing qualified scientific researchers access to anonymized data and clinical study reports from the company’s clinical trials for the purpose of conducting legitimate scientific research. MSD is also obligated to protect the rights and privacy of trial participants and, as such, has a procedure in place for evaluating and fulfilling requests for sharing company clinical trial data with qualified external scientific researchers. The MSD data sharing website (available at: http://engagezone.msd.com/ds_documentation.php) outlines the process and requirements for submitting a data request. Applications will be promptly assessed for completeness and policy compliance. Feasible requests will be reviewed by a committee of MSD subject matter experts to assess the scientific validity of the request and the qualifications of the requestors. In line with data privacy legislation, submitters of approved requests must enter into a standard data-sharing agreement with MSD before data access is granted. Data will be made available for request after product approval in the US and EU or after product development is discontinued. There are circumstances that may prevent MSD from sharing requested data, including country or region-specific regulations. If the request is declined, it will be communicated to the investigator. Access to genetic or exploratory biomarker data requires a detailed, hypothesis-driven statistical analysis plan that is collaboratively developed by the requestor and MSD subject matter experts; after approval of the statistical analysis plan and execution of a data-sharing agreement, MSD will either perform the proposed analyses and share the results with the requestor or will construct biomarker covariates and add them to a file with clinical data that is uploaded to an analysis portal so that the requestor can perform the proposed analyses.
